# Gastrotomy

**Published:** 1890-03

**Authors:** E. S. Lawley


### GASTROTOMY.

By E. S. Lawley.

During the past Summer the following case came under my notice while with Mr. Wende, of Buffalo, N. Y.

A cross-bred, short-haired dog, weighing about forty pounds, was brought to the infirmary by a boy who was in great distress, the animal having swallowed a silver dollar, so he said.

I administered an emetic (tartar emetic, 4 grs.). Vomition took place soon after, but no silver dollar came to sight, so I advised the boy to take his dog home and watch carefully in case he passed it with his feces. Nearly six weeks afterwards, viz., on the 10th of June, the boy and dog reappeared, the latter looking as though he had undergone severe trials, being very much emaciated, and passing blood and mucus per rectum. The boy informed us that the dog had nearly lost its appetite, and what little food he would take was invariably ejected; this led us to suppose there might be something in the dollar theory after all, of which we had at first been rather sceptical. The dog being a great pet, and the boy most anxious to see something done to relieve its most evident suffering, we decided to operate on him and remove the coin if possible.

Not thinking it necessary to diet the animal, it being then in low condition, we proceeded to operate.

The instruments used were scalpels with metal handles, artery forceps, scissors, three needles, two having different sized carbolized gut in them, the third strong cotton (prepared). The instruments were placed in a weak solution of carbolic acid. The hands of the operator were thoroughly scrubbed in hot water and soap, then swilled in a weak solution of carbolic acid. The attendant then put the animal on a small operating table used for spaying bitches, and administered an anæsthetic, viz., sulph. ether. As soon as this had taken effect, the animal was turned on its back, the abdomen sponged and shaved with an ordinary razor, then thoroughly sponged and irrigated with a 1 to 1000 per cent. solution of corrosive sublimate. An incision about 3½ inches long, parallel with and through the linea albæ, exposed the intestines, which were now carefully examined by manipulation, then the stomach, but no silver dollar so far. We were beginning to get disheartened, when something hard was felt about two inches in the duodenum, which proved to be the truant coin. After carefully working it back into the stomach, the latter was drawn out of the opening, exposing the greater curvature, an incision made parallel with the blood supply, the contents and dollar squeezed out well over the side to prevent any getting in among the intestines and causing any irritation; well irrigated, and about five stitches put in with the fine gut; again irrigated and returned, this time with a solution of boric acid; the intestines, with which we thought our hands had come in contact, were also sponged with the latter; the abdominal muscles were sutured with the stouter gut, the common integument with the cotton. The animal was laid near the open door, and a small quantity of cold water dashed on its head. It came round within the course of five minutes, and within half an hour was able to be taken home. It vomited a small quantity of blood, but seemed otherwise all right. The boy and dog left for home, the former having instructions not to give the animal anything but water for twenty-four hours, and to keep it tied up.

Five days after the dog was brought back, still unable to retain food on its stomach, so ordered sub-nitrate of bismuth 10 grs. three times a day on an empty stomach, and to be fed on peptonized beef and given 5 min. of tincture of nux vomica three times a day after meals. The dog continued to improve, and when I left Buffalo in September was looking as fat and sleek as a seal.

